# Directed evolution and targeted mutagenesis to murinize *listeria monocytogenes *internalin A for enhanced infectivity in the murine oral infection model

**DOI:** 10.1186/1471-2180-10-318

**Published:** 2010-12-13

**Authors:** Ian R Monk, Pat G Casey, Colin Hill, Cormac GM Gahan

**Affiliations:** 1 Alimentary Pharmabiotic Centre & Department of Microbiology, University College Cork, Western Road, Cork, Ireland; 2 Alimentary Pharmabiotic Centre, Department of Microbiology & School of Pharmacy, University College Cork, Western Road, Cork, Ireland; 3 School of Genetics and Microbiology, Department of Microbiology, Trinity College Dublin, Ireland

## Abstract

**Background:**

Internalin A (InlA) is a critical virulence factor which mediates the initiation of *Listeria monocytogenes *infection by the oral route in permissive hosts. The interaction of InlA with the host cell ligand E-cadherin efficiently stimulates *L. monocytogenes *entry into human enterocytes, but has only a limited interaction with murine cells.

**Results:**

We have created a surface display library of randomly mutated InlA in a non-invasive heterologous host *Lactococcus lactis *in order to create and screen novel variants of this invasion factor. After sequential passage through a murine cell line (CT-26), multiple clones with enhanced invasion characteristics were identified. Competitive index experiments were conducted in mice using selected mutations introduced into *L. monocytogenes *EGD-e background. A novel single amino acid change was identified which enhanced virulence by the oral route in the murine model and will form the basis of further engineering approaches. As a control a previously described EGD-InlA^m ^murinized strain was also re-created as part of this study with minor modifications and designated EGD-e InlA^m^*. The strain was created using a procedure that minimizes the likelihood of secondary mutations and incorporates *Listeria*-optimized codons encoding the altered amino acids. *L. monocytogenes *EGD-e InlA^m^* yielded consistently higher level murine infections by the oral route when compared to EGD-e, but did not display the two-fold increased invasion into a human cell line that was previously described for the EGD-InlA^m ^strain.

**Conclusions:**

We have used both site-directed mutagenesis and directed evolution to create variants of InlA which may inform future structure-function analyses of this protein. During the course of the study we engineered a murinized strain of *L. monocytogenes *EGD-e which shows reproducibly higher infectivity in the intragastric murine infection model than the wild type, but does not display enhanced entry into human cells as previously observed. This murinized *L. monocytogenes *strain will provide a useful tool for the analysis of the gastrointestinal phase of listeriosis.

## Background

**Figure 1:**
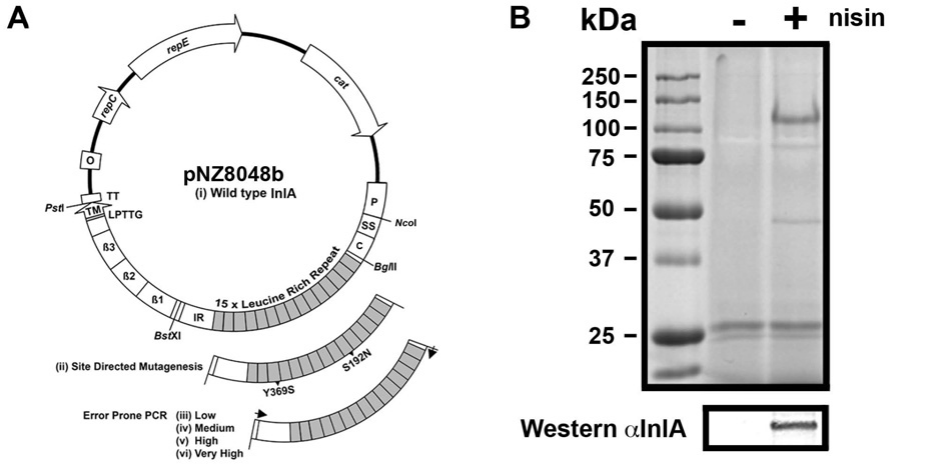
**Nisin inducibe InlA plasmid constructs and the expression of InlA^WT ^on the surface of *L. lactis***. **A**. Lactococcal nisin inducible plasmid pNZB with the entire (i) *inlA*^WT ^gene from EGD-e cloned upstream of the nisin inducible *nisA *promoter (P). The labels for the InlA domains are described in the introduction text. The naturally occuring *Bgl*II/*Bst*XI restriction sites within the *inlA *gene encompass the entire LRR required for the interaction with E-cadherin. These two sites were used for the cloning of the (ii) the murinized version of *inlA *(inlA^m^*) with the amino acid changes as described by Wollert *et al*. [17] (created by site directed mutagenesis) and (iii) four randomly mutated banks of *inlA *generated by error prone PCR. **B**. A lysozyme cell wall extract was isolated from *L. lactis *InlA^WT ^grown under the conditions used for invasion assay. Exponential phase cells (OD = 0.6) were cultured for 1 h in the presence (+) or absence (-) of nisin (approx 10 ng/ul). Proteins were run on 10% SDS-PAGE and either stained with coomassie blue or blotted and detected with a InlA monoclonal antibody [23].

Internalin A (InlA) is a sortase achored, cell wall protein and a critical factor in the pathogenesis of the foodborne Gram-positive pathogen *Listeria monocytogenes*. InlA stimulates *L. monocytogenes *entry into normally non-phagocytic intestinal enterocytes [1]. The protein is 800 amino acids (aa) in length and composed of seven distinct domains (Figure 1a); (i), 1-35 aa: a consensus N-terminal signal sequence (SS); (ii), 35-78 aa: forms a cap at the N-terminus tip of mature protein (C); (iii), 79-407 aa: 15 Leucine rich repeats (LRR) with 14 containing 22 aa (repeat 6 contains 21 aa) (shaded grey); (iv), 415-495 aa: an inter-repeat domain (IR); (v), 518-706 aa: three β-repeat domains, which may serve as a stalk to project the sickle shaped LRR out from the cell surface (β1, β2 and β3); and (vi), 767-771 aa: a sortase cleavage site (LPPTG) for peptidoglycan cross linking and (vii) 771-800 aa: a membrane targeting sequence (TM)[2]. Domain (iii) containing the LRR's of InlA is sufficient to stimulate enterocyte uptake [3,4]. The enterocyte ligand for InlA was identified as E-cadherin (CDH1) [5], which is required by host cells for the formation of tight junctions and to promote cellular polarization, communication and differentiation [6]. The localization of CDH1 on the basolateral face of differentiated cells suggested that invasion was a secondary event, occurring after non-specific uptake by M cells [5]. Oral infection studies using rats [7] and mice [8] provided support for this hypothesis. However, oral infections resulted in the invasion of enterocytes in a guinea pig model [9]. Human colonic Caco-2 enterocyte cells are also directly permissive to infection *in vitro *[9,10]. These seemingly anomalous results are due to the reduced affinity of murine CDH1 (mCDH1) for InlA. The reduced affinity was localized to amino acid 16 which is a proline in guinea pig and human CDH1 (hCDH1) but in rats and mice a glutamic acid is present [11]. This discovery led to the development and application of a transgenic mouse model expressing both human and murine CDH1 within intestinal enterocytes, which conclusively demonstrated the role of InlA in the pathogenesis of orally acquired *L. monocytogenes *[12]. In an elegant study, the site of enterocyte cell extrusion at the tips of intestinal villi was identified as a mechanism for exposing CDH1 on the apical surface at multicellular junctions [13]. More recently, a transgenic mouse strain that ubiquitously expresses human E-cadherin has been developed to demonstrate a role for InlA (and InlB) in fetoplacental listeriosis [14].

The crystal structure of InlA in complex with hCDH1 demonstrated the structural importance of proline 16 for the interaction [15]. *In silico *analysis confirmed that the reduced affinity of InlA for mCDH1was essentially due to the steric hindrance imposed by the bulky glutamic acid at aa 16, which therefore could not interact with the hydrophobic pocket (between LRR's 5, 6 and 7 of InlA) created by the removal of one amino acid from LRR 6 [15]. Overall the crystal structure identified 28 residues of hCDH1 that interact with the residues across the LRR region. Structural data and the invasion results from previous research [3,4] have confirmed the essential nature of the LRR's in the InlA::CDH1 interaction.

Small animal model of listeriosis have a number of significant limitations. Even though rabbits and guinea pigs possess a permissive CDH1, they have recently been shown to be resistant to systemic infection due to a species specificity observed in the InlB/host interaction [16]. InlB is required for efficient hepatocyte/endothelial cell invasion in the mouse model and in certain human cell lines. A novel approach to address the lack of appropriate animal models focused on the 'murinization' of *L. monocytogenes *rather than the 'humanization' of mice [17]. Rational protein design based on the structural data of the InlA/hCDH1 complex, identified two mutations in InlA (Ser192Asn and Tyr369Ser) that dramatically increased the affinity for both hCDH1 and mCDH1. This allowed the development of a variant of *L. monocytogenes *EGD-e (EGD-InlA^m^) capable of establishing systemic infections in C57BL/6J mice after oral inoculation [17]. However, the strain also exhibited a 2-fold increase in adhesion and consequently invasion into human cells, suggesting that the alteration in tropism towards mice also could enhance the virulence towards humans.

To address any remaining concerns regarding human virulence of murinized *L. monocytogenes*, we conducted random mutagenesis of InlA combined with surface display on a non-invasive, Gram-positive, *Lactococcus lactis *to identify mutations that improve the entry into a colonic murine cell line. Using the CT-26 cells as a selection tool, multiple positive mutations in independent clones were identified with an enrichment in the InlA/hCDH1 interacting residues. The *inlA *genes from 4 *L. lactis *clones were separately recombined into the *inlA *chromosomal locus in EGD-eΔ*inlA *generating EGD-e A to D. Also, a version of EGD-InlA^m ^[17] was created in order to permit comparison with our newly generated InlA mutant strains. In contrast to the strategy employed by Wollert et al. [17] we utilised preferred *Listeria *codons for the mutated 192Asn and 369Ser and designated the strain; EGD-e InlA^m^*. Strains were competed against EGD-e InlA^m^* in oral murine competitive index assays [18]. A novel aa mutation was identified which enhanced InlA/mCHD1 interaction compared to EGD-e. In agreement with earlier studies [17] the adherence/invasion into Caco-2 cells and virulence by murine intravenous infection of the codon-optimized EGD-e InlA^m^* strain was indistinguishable from EGD-e, while EGD-e InlA^m^* alone exhibited highly reproducible murine oral infections.

## Methods

### Bacterial and Cell Culture

Bacterial strains, plasmids and oligonucleotides are described in Table 1. For the routine propagation of *L. lactis *MG1363 derivative NZ9000, cells were grown statically at 30°C in M17 (Oxiod) broth containing 0.5% w/v filter sterilized glucose (GM17). *L. monocytogenes *were cultivated in BHI (Oxiod) and *Escherichia coli *grown in LB at 37°C with shaking at 200 rpm. For growth on agar, respective broths were solidified with 1.5% (w/v) agar (Merck). For blue/white screening in *L. monocytogenes*, X-gal (Merck) was incorporated into BHI agar at 100 μg/ml. Antibiotics were added when required: erythromycin *E. coli *- 250 μg/ml, *L. monocytogenes *- 5 μg/ml and chloramphenicol *L. lactis *- 5 μg/ml. Plasmids were isolated from NZ9000 after overnight growth in 10 ml of GM17. To lyse, the pellet was resuspended in 500 μl of P1 buffer (see Qiagen manual) containing 30 μg of lysozyme and incubated for 30 min at 37°C. The lysate was processed as described in the Qiaprep spin miniprep kit (Qiagen). A nisin filtrate for P*nisA *induction was isolated from the supernatant of an overnight *L. lactis *culture of NZ9700 (filter sterilized through 0.22μM low protein binding filters - Millipore), aliquots frozen at -20°C. For all InlA induction experiments, overnight *L. lactis *NZ9000 cultures (containing pNZ8048 plasmids) were diluted 1:20 in 10 ml of fresh GM17 and grown to an OD_600 nm _of 0.5 (approximately 2 h). The expression of *inlA *was induced with 10 μl of nisin and grown for a further hour to an OD = 1.0 (5×10^8 ^cfu/ml). The murine (CT-26) and human (Caco-2) colonic epithelial cell lines were routinely cultured at 37°C in 5% CO_2_. Media was composed of DMEM glutamax, 10% FBS, Pen/Strep and 1% non essential amino acids with all cell culture media purchased from Gibco. Oligonucelotides were purchased from Eurofins MWG Operon.

**Table 1:** Bacterial strains, plasmids and oligonucleotides

Name	Description	Source
**Bacterial strains**		
EC10B	*E. coli *DH10B derivative, with *repA *integrated into the *glgB *gene. Kan^r^.	[20]
NZ9000	Nisin responsive *L. lactis *MG1363 derivative, with *nisRK *integrated into the *pepN *gene.	[26]
EGD-e	*L. monocytogenes *1/2a strain. Genome sequenced. Obtained from Werner Goebel.	[39]
EGD-eΔ*inlA*	EGD-e with the E-cadherin interacting region of InlA deleted (amino acids 80 to 506)	[20]
EGD-eΔ*inlA*::pIMK2*inlA*	EGD-e Δ*inlA *with InlA over expressed from the Phelp promoter integrated at tRNA^Arg ^locus, Kan^r^	[20]
EGD-e InlA^m^*	EGD-e with *inlA *residues S192N and Y369 S modified in the chromosome.	This study
EGD-e A	EGD-eΔ*inlA *with *inlA *locus recreated containing SDM change N259Y in the chromosome.	This study
EGD-e B	EGD-eΔ*inlA *with *inlA *locus recreated containing SDM change Q190L in the chromosome.	This study
EGD-e C	EGD-eΔ*inlA *with *inlA *the locus recreated containing SDM changes S173I, L185F and L188F in the chromosome.	This study
EGD-e D	EGD-eΔ*inlA *with *inlA *locus recreated containing SDM changes T164A, K301I and G303E in the chromosome.	This study
EGD-e InlA^m^* ::pIMC3*ery*	EGD-e InlA^m^* with the IPTG inducible expression of erythromycin integrated in the tRNA^ARG ^locus, Cm^r^.	This study
EGD-e::pIMC3*kan*	EGD-e with the IPTG inducible expression of kanamycin integrated in the tRNA^ARG ^locus, Cm^r^.	[18]
EGD-e A::pIMC3*kan*	EGD-e A with the IPTG inducible expression of kanamycin integrated in the tRNA^ARG ^locus, Cm^r^	This study
EGD-e B::pIMC3*kan*	EGD-e B with the IPTG inducible expression of kanamycin integrated in the tRNA^ARG ^locus, Cm^r^	This study
EGD-e C::pIMC3*kan*	EGD-e C with the IPTG inducible expression of kanamycin integrated in the tRNA^ARG ^locus, Cm^r^	This study
EGD-e D::pIMC3*kan*	EGD-e D with the IPTG inducible expression of kanamycin integrated in the tRNA^ARG ^locus, Cm^r^	This study
NZ9700	Nisin producer, progeny of NIZO B8 and MG1363 (Rif^r ^and Strp^r^) conjugation.	[26]
**Plasmids**		
pNZB	Nisin inducible plasmid with heterologous gene expressed from the *nisA *promoter. *Bgl*II site upstream of *nisA *removed.	This study
pNZB*inlA*^WT^	Internalin A from EGD-e containing the entire gene including signal sequence. Cloned into *Nco*I/*Pst*I of pNZB.	This study
pNZBinlA^m^*	Internalin A containing S192N and Y369 S in pNZB.	This study
pNZB*inlA *Bank-iii	Error Prone PCR with low level of mutation 0-4.5 nt per kb.	This study
pNZB*inlA *Bank-iv	Error Prone PCR with medium level of mutation 4.5-9 nt per kb.	This study
pNZB*inlA *Bank-v	Error Prone PCR with high level of mutation 9-16 nt per kb.	This study
pNZB*inlA *Bank-vi	Error Prone PCR with very high level of mutation 9-16 nt per kb.	This study
pORI280	RepA negative gene replacement vector, constitutive lacZ, 5.3 kb, Em^r^.	[40]
pORI280inlA(SDM)	PCR amplified mutated inlA^m^* into pORI280 as *Nco*I/*Pst*I fragment. Contains wild type inlA promoter.	This study
pORI280*inlA*(A)	PCR amplified mutated *inlA *(from bank v clone 6 containing N259Y) into pORI280 as *Nco*I/*Pst*I fragment. Contains Wt *inlA *promoter.	This study
pORI280*inlA*(B)	PCR amplified mutated *inlA *(from bank iii clone 3 containing Q190L) into pORI280 as *Nco*I/*Pst*I fragment. Contains Wt *inlA *promoter.	This study
pORI280*inlA*(C)	PCR amplified mutated *inlA *(from bank v clone 6 containing S173I, L185F, L188F) into pORI280 as *Nco*I/*Pst*I fragment. Contains Wt *inlA *promoter.	This study
pORI280*inlA*(D)	PCR amplified mutated *inlA *(from bank v clone 8 containing T164A, K301I, G303E) into pORI280 as *Nco*I/*Pst*I fragment. Contains Wt *inlA *promoter.	This study
pVE6007	Temperature-sensitive helper plasmid, supplies RepA in *trans*. Cm^r^.	[41]

**Name**	**Oligonucleotide sequence (5'-3')*^a^***	**Restriction site**

IM194 (inlA-F)	ATAT**CCATGG**AAAAAAACGATATGTATGGTTG	*Nco*I
IM188 (inlA-R)	TTTT**CTGCAG**TTATTTACTAGCACGTGCTTTTTTAG	*Pst*I
IM345 (S192N SDM-F)	CAGGTTTAACTAGTCTACAGCAATTAAATTTTGGTAATCAAGTGACAGATTTAAAACC	
IM346 (S192N SDM-R)	GGTTTTAAATCTGTCACTTGATTACCAAAATTTAATTGCTGTAGACTAGTTAAACCTG	
IM349 (Y369 S SDM-F)	CAAAGCTTCAAAGATTATTTTTCTCTAATAACAAGGTAAGTGACGTAAG	
IM350 (Y369 S SDM-R)	CTTACGTCACTTACCTTGTTATTAGAGAAAAATAATCTTTGAAGCTTTG	
IM490 (Chromosome-F)	ATAT**CCATGG**AAAAGGAGTGTATATA***GTG***AGAAAAAAACGATATGTATGG	*Nco*I
IM466 (Chromosome-R)	ATAT**CTGCAG**CAAACGTTGCTGTATAGCTATTGG	*Pst*I
IM467 (inlA out-F)	TATATAGGAAAAATGTGCTGGAACG	
IM468 (inlA out-R)	TCCTTGATAGTCTACTGCTTGAGTCG	
IM317 (inlA muta-F)	AAAC**AGATCT**AGACCAAGTTACAACG	*Bgl*II
IM318 (inlA muta-R)	AATT**CCA**CTTCTT**TGG**TTGTTTCTTTGC	*Bst*XI

*^a ^*Restriction sites are highlighted in bold. Mutated triplets are underlined. The start codon of *inlA *is in italics.

### Production of electrocompetent *Lactococcus lactis*

The protocol of Holo and Nes [19] was adapted for the transformation of *L. lactis *MG1363 derivative NZ9000. A GM17 overnight culture of NZ9000 was diluted 1:100 into 5 ml of GM17 containing 500 mM sucrose and 2.5% glycine (GS-GM17). This culture was inoculated into 50 ml of fresh GS-GM17 and grown overnight. The 50 ml culture was inoculated into 400 ml of fresh GS-GM17, grown to OD600 of 0.3 and cells were subsequently harvested by centrifugation at 4,000 × g for 20 min at 4°C. The pellet was resuspended in 200 ml of ice cold SGB (500 mM sucrose and 10% (w/v) glucose - filter sterilized), centrifuged, resuspended in 100 ml SGB and left on ice for 15 min. The cells were centrifuged, resuspended in 50 ml SGB and left on ice for 15 min before a final centrifugation and re-suspension with 2 ml SGB. Cells were frozen at -80°C in 40 μl aliquots. To electroporate, cells were thawed on ice, mixed with 4 ul of pellet paint (Novagen) precipitated DNA and transferred to a 1 mm electroporation cuvette (Biorad). Cells were pulsed at 20 kV/cm, 200 Ω and 25 μF, regenerated in 1 ml GM17 containing 2 mM CaCl2/20 mM MgCl2 for 1.5 h and then plated onto GM17 agar containing 5 μg/ml chloramphenicol. An efficiency of 1 × 10^7 ^cfu/μg was routinely obtained with pNZ8048.

### Cloning of InlA into pNZB

The unique *Bgl*II site up stream of the *nisA *promoter in pNZ8048 was removed by linearization of the vector with *Bgl*II and ends blunted with T4 DNA polymerase. The vector was religated to generate pNZB. The *inlA *gene was PCR amplified (primers IM194 and IM188) as described previously [20], digested with *Nco*I/*Pst*I and ligated into the complementary digested pNZB. Ligations were directly electroporated into NZ9000 as described above and the sequence of the *inlA *gene was verified by DNA sequencing.

### QuikChange mutagenesis in *L. lactis*

Primers for site directed mutagenesis (SDM) (Table 1) were designed according to the Quikchange SDM manual (Stratagene). All plasmid template isolated from NZ9000 strains was methylated with Dam methylase following manufacturer recommendations (New England Biolabs). The PCR thermocycling conditions were conducted as described previously [21]. Separate 50 μl KOD hotstart high fidelity polymerase PCR reactions were preformed with each primer for 10 cycles and an extension time of 5 min 30 sec. After 10 cycles the reactions were combined and continued for an additional 18 cycles. Amplimers were column purified (Qiaquick PCR purification kit, Qiagen) and digested overnight with *Dpn*I (Roche). Digests were pellet paint precipitated and the half of the digest directly electroporated into NZ9000. Between 200 and 1000 colonies were obtained per transformation. The protocol was repeated to combine SDM changes. From the final mutagenized plasmids, *Bgl*II/*Bst*XI fragments containing the LRR region of InlA were excised and ligated into complementary digested pNZB*inlA*^WT^.

### Isolation of cell wall proteins

Cell wall proteins were isolated from nisin induced 10 ml NZ9000+pNZB*inlA*^WT ^culture as described by previously [22], except cells were rendered as protoplasts for 1 hr at 30°C without mutanolysin. Blotted proteins were probed with the InlA specific monoclonal antibody described by Hearty *et al *[23].

### Random Bank of *inlA *mutants in NZ9000

The generation of a randomly mutated *inlA *bank between amino acids 74 and 512 (containing the LRR) of InlA was accomplished by error prone PCR with Mutazyme II (Stratagene). Plasmid DNA (pNZB*inlA*^WT^) was used as template in the reaction (primers IM317 and IM318) and a 1.3 kb fragment amplified between two naturally occurring restriction sites (*Bgl*II and *Bst*XI). From the mutagenesis reactions, four different mutation rates by varying the amount of template used ((iii) 1000 ng (iv) 250 ng (v) 10 ng and (vi) 0.1 ng). This equates to a sliding scale of increasing mutation frequency. Each amplimer pool was digested with *Bgl*II and *Bst*XI and ligated into complementary digested pNZ8048b*inlA*. The ligations (100 ng of pNZB with 240 ng of *inlA*) were pellet paint precipitated and electroporated into electrocompetent NZ9000 (repeated twice). For each pool a total of 40,000 colonies were obtained with plasmid religations accounting for 0.125% of the total (about 50 colonies per 40,000). The colonies from each mutation frequency were pooled and frozen at -80°C. From each mutation frequency, 10 individual colonies were subjected to plasmid isolation as described above and the mutated region sequenced to access the level of mutagenesis.

### CT-26 and Caco-2 invasion assays

Overnight cultures of NZ9000 containing pNZB only or pNZB*inlA *derivatives (Figure 1a) were induced as described above. A one ml aliquot was then pelleted at 4,000* × g *for 5 min and resuspended in 1 ml of DMEM. Cells were centrifuged, resuspended in fresh DMEM and then diluted to a multiplicity of infection of 25:1. *L. monocytogenes *cells were grown as described previously prior to invasion [20]. CT-26 [24] and Caco-2 cells were seeded at 2 × 10^4 ^and 1 × 10^5 ^cells, respectively and grown for 4 days until confluency in 24 well plates (Falcon). On the day prior to use, antibiotics were removed from the media. On the day of use, cells were washed twice with DMEM to remove FBS. Both cell types were invaded for 1 h at 37°C in 5% CO_2_, washed once with Dulbecco's PBS (Sigma) and then overlayed with DMEM containing 10 (Caco-2) or 100 μg/ml (CT-26) gentamicin for 1 h. Monolayers were washed a further three times with PBS to remove residual antibiotic and then lysed with 1 ml of ice cold sterile water. Bacterial cells were enumerated by serial dilution in PBS and plated on GM17 agar containing 5 μg/ml chloramphenicol. The remaining lysate from error prone PCR pools were inoculated into GM17 containing 5 μg/ml chloramphenicol, grown overnight, stocked at -80°C with the protocol repeated for seven passages through CT-26 cells. EGD-e derivatives were plated onto BHI agar.

### Internalin A chromosomal mutagenesis in *L. monocytogenes*

A 2 kb fragment was PCR amplified (primers IM467 and IM490) from the appropriate mutated pNZ8048b*inlA *plasmid, with primer design incorporating the first 16 nt upstream of the *inlA *GTG start codon. The amplimers were digested with *Nco*I/*Pst*I, ligated into complementary digested pORI280 and transformed into *E. coli *strain EC10B (Table 1). The plasmids pORI280 and pVE6007 we co-transformed into EGD-eΔ*inlA *and mutagenesis preformed as described by previously [20]. The reconstruction of the *inlA *locus was identified by colony PCR (primers IM317 and IM318) with the integrity of the gene confirmed by DNA sequencing.

### Intragastric versus intravenous infections of Balb/c mice

For all murine experiments, 6-8 week old female Balb/c mice (Harlan) were used. All experiments were approved by the institutional ethics committee. Tail vein intravenous infections were conducted as described previously [18] with an inoculum comprised of equal numbers of EGD-e::pIMC3*kan *and EGD-e InlA^m^* ::pIMC3*ery *(2 × 10^4 ^total in 100 μl). For oral inoculation, overnight cultures were centrifuged (7,000 × g for 5 min), washed twice with PBS and resuspended at 5 × 10^10 ^cfu/ml in PBS containing 100 mg/ml of CaCO_3_. A 200 μl inoculum was comprised of either a single strain (5 × 10^9 ^cfu) or a two strain mixture (5 × 10^9 ^of each strain). Mice were intragrastrically gavaged and the progression of infection followed over a three day time course. For bioluminescent imaging, mice were anesthetized on day 1 through to day 3 with isoflurane gas and imaged in a Xenogen IVIS 100 (Xenogen) at a binning of 16 for 5 min. Mice were euthanized with spleen and livers aseptically removed, imaged (binning of 8 for 5 min) and enumerated as previously described [18].

## Results

### A *L. monocytogenes *gentamicin protection assay for murine cells

**Figure 2:**
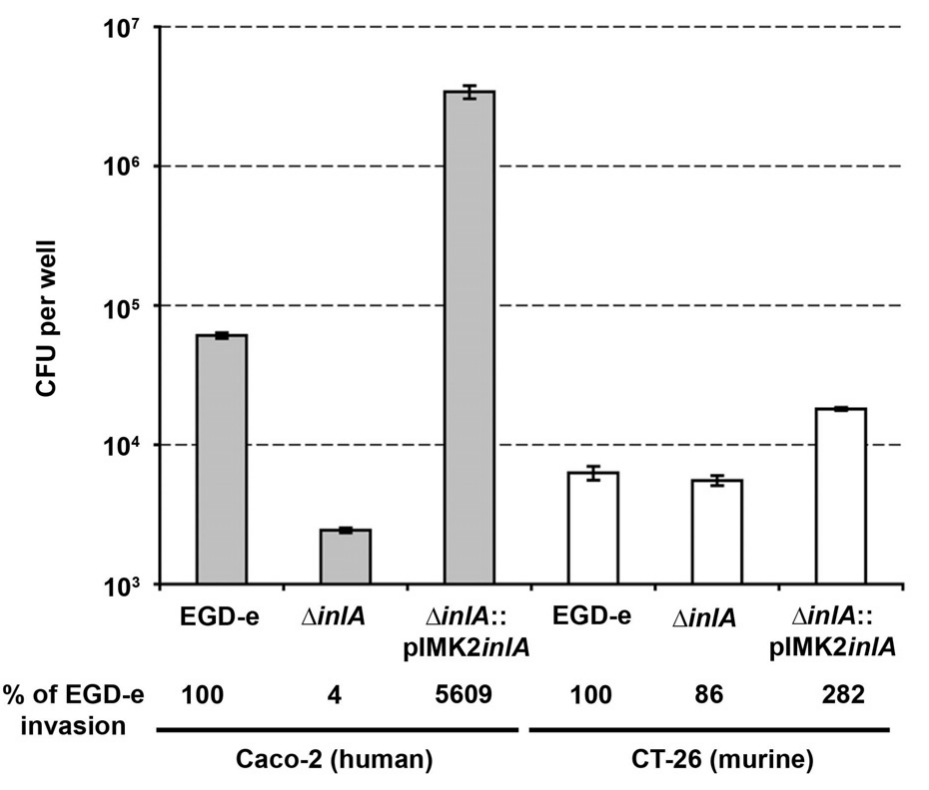
**InlA dependent invasion of EGD-e derrived strains into human (Caco-2: grey bars) or murine (CT-26: white bars) monolayers**. Exponential phase *L. monocytogenes *cells (OD = 0.8) were invaded (MOI of 25:1) in triplicate for 1 h before overlaying with gentamicin. Invasion was expressed as the average cfu count per well (with standard deviation) or invasion relative to EGD-e (below graph) (n = 3). The graph is representative of the data from three independent experiments.

Invasion into Caco-2 cells by *L. monocytogenes *is dependent on the expression of functional InlA [10]. We confirmed that a *L. monocytogenes *mutant producing InlA without the LRR and IR domain (Δ*inlA*) is severely compromised in invasion, while an over expressing InlA strain exhibits dramatically enhanced invasion (Figure 2). To establish an equivalent murine assay for *L. monocytogenes *we used monolayers of CT-26 cells (murine colonic carcinoma cell line) originally isolated from Balb/c mice chemically treated to induce tumor formation [24]. While CT-26 cells are not enterocyte like (they exhibit an undifferentiated-fibroblast appearance [25]), the results from invasion assays showed that they provide characteristics suitable for use as an invasion model (Figure 2). The Δ*inlA *strain displayed a slight reduction (not statistically significant) in invasion compared to EGD-e, while over expression of InlA resulted in a modest increase in invasion. We speculate that this is due to a reduced affinity of InlA for mCDH1, however we have not assayed for mCDH1 production by CT-26 cells.

**Figure 3:**
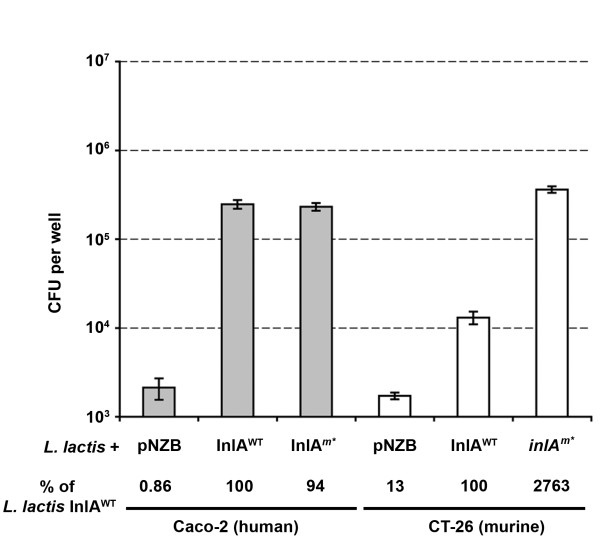
**Invasion of *L. lactis *expressing wild type or murinized InlA into Caco-2 (grey bars) or CT-26 (white bars) monolayers**. Nisin induced *L. lactis *cells were invaded (MOI of 25:1) for 1 h before overlaying with gentamicin. Invasion was expressed as average cfu count (with standard deviation) or invasion relative to *L. lactis *plasmid only (below graph) (n = 3). The graph is representative of the data from three independent experiments.

Heterologous expression was then employed to distinguish InlA from additional virulence determinants on the surface of the *L. monocytogenes*. We chose to use the well characterized nisin inducible expression system [26] (Figure 1) to produce full length InlA on the surface of *L. lactis*. The system was chosen because production of functional InlA on the cell surface of *L. lactis *had previously been documented [27]. We compared the entry of *L. lactis *containing vector only (*L. lactis*-pNZB), producing wild type InlA (*L. lactis *InlA^WT^) or producing InlA containing the Ser192Asn and Tyr369Ser, but with different codon usage to the previously described murinized InlA^m ^[17] (*L. lactis *InlA^m^*) into Caco-2 and CT-26 cells. The presence of InlA on the cell surface was confirmed by Western blot analysis (Figure 1b). The level of invasion for *L. lactis*-pNZB into Caco-2 cells is similar to that observed for EGD-eΔ*inlA *(Figure 2 and 3). As *L. lactis *is non invasive, the surviving bacterial cells probably represent bacteria not killed by the gentamicin treatment rather than internalized cells, as documented previously [1]. A similar level of entry into Caco-2 cells was observed for *L. lactis *InlA^WT ^and *L. lactis *InlA^m^*, while entry into CT-26 cells was 27-30 fold greater for *L. lactis *InlA^m^* compared to *L. lactis *InlA^WT ^(Figure 2).

In contrast to a previous report [11], we observed an increased invasion into a murine cell line by the *L. monocytogenes *strain over-expressing InlA^WT ^in contrast to the plasmid only control (Figure 2). A similar trend was observed when the *L. monocytogenes *InlA over-expressing strain and Δ*inlA *strain were compared (Figure 2) and was also seen in experiments in the *L. lactis *background (Figure 3). These results could be due to the high level of *inlA *expression from the P*nis *and P*help *promoters, amplifying the differences in InlA on the surface of *L. lactis *and *L. monocytogenes *cells (Figure 2 and 3). We interpret these results as evidence of a specific interaction between InlA and a cell surface receptor on CT-26 cells which stimulates bacterial cell entry. To summarise, we have established a gentamicin protection assay, capable of discriminating InlA mediated invasion into a murine cell line.

### Generation and screening of a random bank of InlA LRR mutants

**Figure 4:**
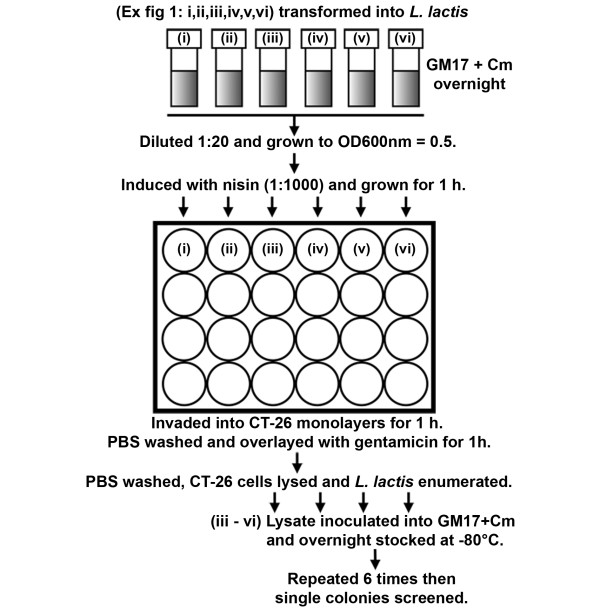
**Enrichment protocol for the selection of mutations in InlA conferring enhanced invasion of *L. lactis *into CT-26 cells**. Cultures of *L. lactis *+ pNZB containing (i) *inlA*^WT ^(ii) inlA^m^* or (iii-vi) 4 banks of clones with different levels of mutation in the LRR of *inlA*^WT ^were induced with nisin and assayed for invasion into CT-26 cells by gentamicin protection assay. The residual lysate from invaded CT-26 cells inoculated into GM17 containing Cm 5 μg/ml and grown overnight, the culture was then frozen at -80°C. The entire process was repeated with the frozen stock serving as the seed for the inoculum.

**Figure 5:**
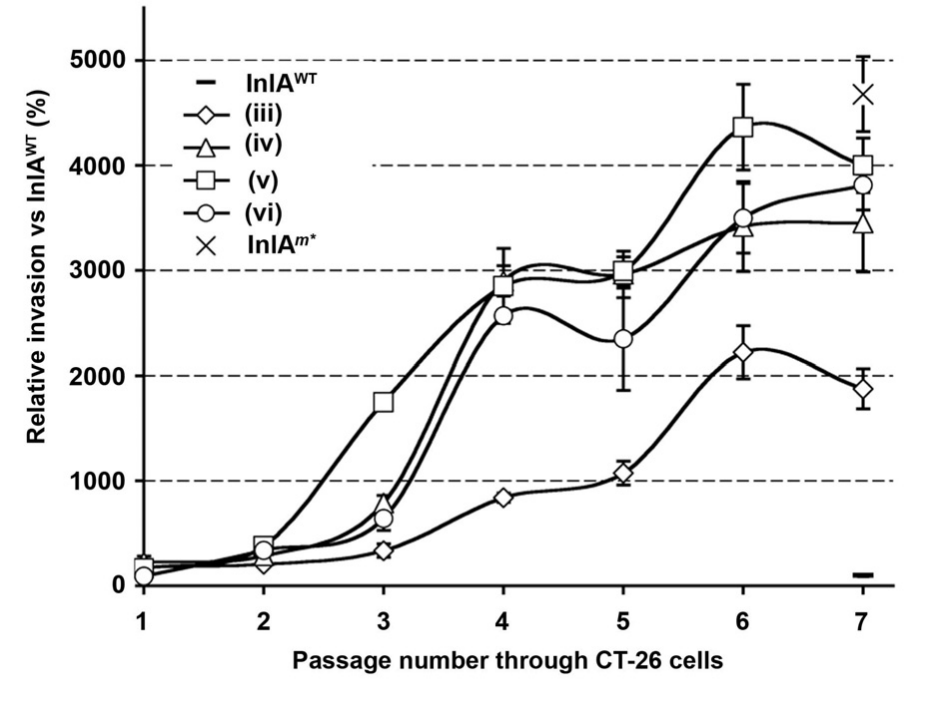
**Enrichment of pools with enhanced invasion into CT-26 cells**. Glycerol stocks from the *L. lactis *banks (both pre and post enrichment passages-including controls: InlA^WT ^and InlA^m^* expressing *L. lactis*) were incoulated into GM17 media. Nisin induced cultures were invaded into CT-26 monolayers. Invasion was expressed relative to *L. lactis *InlA^WT ^(set as 100 percent). The graph is of the data from one experiment.

**Figure 6:**
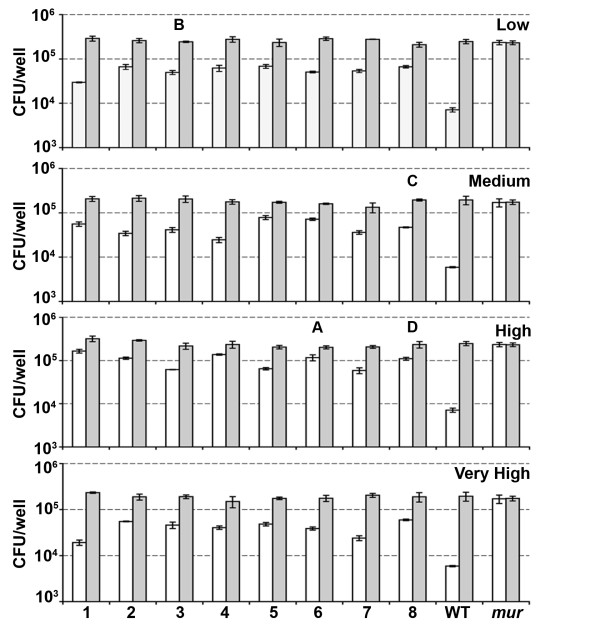
**Invasion attributes of individual *L. lactis *clones post CT-26 enrichment (passage 6) into Caco-2 (grey bars) or CT-26 (white bars) cells**. From each of the four banks, eight clones were picked and invaded with invasion expressed as the average (with standard deviation) from triplicate wells. Sequnce data of the clones is presented in Table 2. Letters above bars indicate sequences that were subsequently used to recreate into the *L. monocytogenes *chromosome. The controls InlA^WT ^(WT) and InlA^m^* (*mur*) expressing *L. lactis *were included for comparison. The graph is of the data from one experiment.

To generate diversity within the *inlA *gene we applied error prone PCR to the LRR region (between naturally occurring *Bgl*II/*Bst*XI sites - Figure 1a). Four separate banks were created containing different levels of mutation frequency, each containing about 40,000 *L. lactis *clones. Initial assessment by DNA sequencing of ten clones from each bank identified mutations throughout the LRR region with the level of mutation correlating with the concentration of input template DNA for the error prone PCR (data not shown). To identify positive mutations, pools were invaded through CT-26 cells *en masse *as detailed in Figure 4. Sequential passages through CT-26 cells were required to remove the background functional InlA from the pools (Figure 5). Of the four banks only the highest mutation frequency resulted in an initial recovery below that of wild type InlA, which suggested that a significant number of clones contained inactivating mutations. From passage two through six a significant enrichment in positive mutations was observed, with a leveling off at passage seven (Figure 5). From passage six, eight clones from each bank were sequenced (Table 2) and assayed individually using both CT-26 and Caco-2 cells (Figure 6). All clones exhibited enhanced entry into CT-26 cells while no apparent differences for cell entry into Caco-2 cells were observed (compared to *L. lactis *InlA^WT^). However, no clones were identified which were capable of matching the level of *L. lactis *InlA^m^* mediated entry into the murine cells. Sequence analysis revealed that 23 of the 32 clones contained amino acid changes in residues involved in direct interaction with CDH1. Of the four banks, only the lowest mutation frequency contained multiple clones with the same mutation (Gln190Leu), with this single amino acid change also found in one clone from an additional bank (Table 2).

**Table 2:** Supplementary information for Figure 6.

Clone	1	2	3	4	5	6	7	8
**(iii) Low**	T273I	**Q190L**	***Q190L***	**Q190L**	**Q190L** T229P	G303E	**Q190L**	**Q190L** N386I

**Fold increase vs Wt**	9.44	5.82	***6.98***	4.15	13.23	12.12	6.10	7.94

**(iv) Medium**	T164A **K301I** G303E T399I	L86F N143K P159A Q196L K218M V224A G303E Q306H	**Q190L** L329Q S470C	T164A **K301I** G303E	**N259Y** T399I	**Q190L** G248R	F193Y **K301E** N413Y K507I	***T164A*** ***K301I*** ***G303E***

**Fold increase vs Wt**	3.25	9.31	7.79	6.85	8.14	6.57	4.05	***10.08***

**(v) High**	L149M **N259Y**	**Q190L** S223C N252Y I351T	S173I G303E T446A D449H	S173I T268I G303E T446A D449H	**Q190L** S223C N252Y I351T	***N259Y***	N239D S311C **N325D**	***S173I*** ***L185F*** ***L188I***

**Fold increase vs Wt**	23.21	15.89	8.64	19.31	9.08	***16.36***	8.24	***15.42***

**(vi) Very High**	**Q190L** A270G **K301G**	V123A **Q190L** P290Q N349D	**Q190L**	Q196K P290S L404S N413Y D457V	N130I **F150V** L203F **Y369F** N381I S487N	L294V S308R **Y369S** N381I S487N	L122I S292T E330V I458V	**Q190L** D199V S377N P444S K495N

**Fold increase vs Wt**	4.14	9.33	6.96	8.71	9.56	7.12	7.51	9.33

Mutations identified in the *Bg*lII/*Bst*XI fragment of pNZB*inlA *(iii-vi) and the invasion increase into CT-26 cells versus *L. lactis *InlA^WT^. The amino acid mutations identified which involved in the interaction between InlA^WT ^and hCDH1 are highlighted in **bold**.

Details highlighted in bold and italics are mutations recombined in the chromosome of EGD-e.

*L. lactis *InlA site directed mutants with fold invasion increase into CT-26 cells vs *L. lactis *InlA^WT ^in brackets: S192N (21), Y369 S (20), S192N+Y369 S (30).

Below: Amino acids in InlA^WT ^which interact with hCDH1 and amino acid changes identified from error prone PCR screen. R85, N104: D Q*, N107, **F150: V**, E170, E172: T*, **Q190: L**, S192, R211, D213, I235, T237, E255, **N259: Y**, **K301: I E G**, N321: Y, E323, **N325: D**, E326, Y343, T345, Y347, F348, R365, F367, **Y369: F S**, W387, S389. * N104 and E172 mutations were found from additional screens and sequencing.

### Characterization of murinized *L. monocytogenes*: competitive index assays

**Figure 7:**
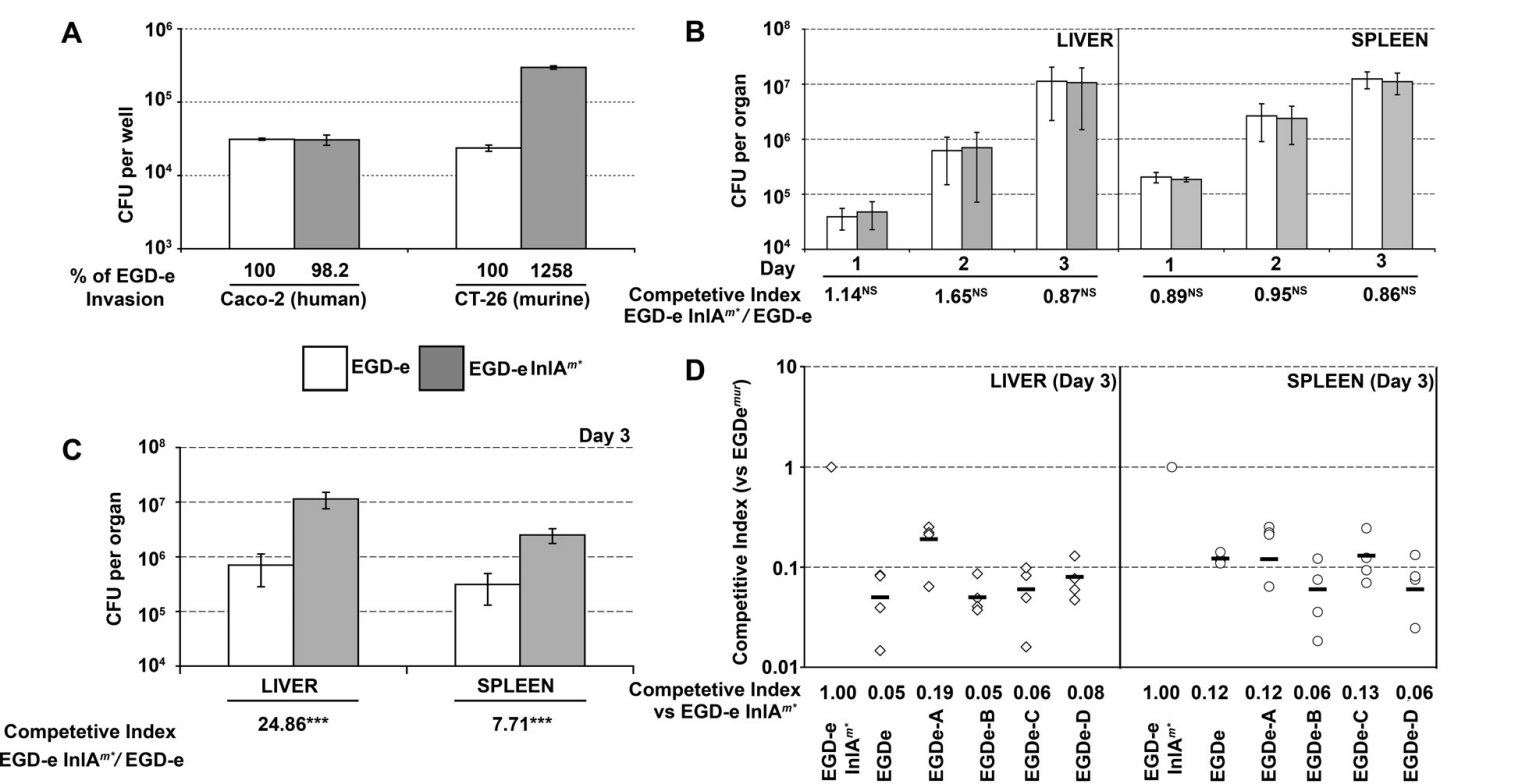
**Recretion of selected InlA mutations in EGD-e**. **A**. Comparison of the invasion attributes of EGD-e and EGD-e InlA^m^* (Ser192Asn/Trp369Ser). Exponential phase *L. monocytogenes *cells (OD = 0.8) were invaded (MOI of 25:1) in triplicate for 1 h before overlaying with gentamicin. Invasion was expressed as the average cfu count per well (with standard deviation) or invasion relative to EGD-e (below graph). The graph is representative of the data from three independent experiments. **B**. The relative virulence of EGD-e compared against EGD-e InlA^m^* (tagged with pIMC3*kan *and pIMC3*ery *respectively) was accessed by competitive index after i.v. infection (1 × 10^4 ^cfu of each strain) of 15 Balb/c mice. On each subsequent day 5 mice were euthanized and spleens and livers aseptically removed and enumerated. Data are presented as the mean and standard deviation of 5 mice, competitive indices and statistical analysis was conducted using the one sample t test as described previously [18]. NS = Not significant. **C**. Oral inoculations of Balb/c mice with EGD-e::pIMC3*kan *and EGD-e InlA^m^* ::pIMC*ery *mixed at a 1:1 ratio in a total inoculum of 1 × 10^10 ^cfu/200 μl containing 100 mg of CaCO_3_. *** = p < 0.005. **D**. Competitive index virulence in a Balb/c oral infection model with EGD-e InlA^m^* ::pIMC3*ery *competed against EGD-e::pIMC3*kan*, EGD-e A::pIMC3*kan *(InlA-N259Y), EGD-e B::pIMC3*kan *(InlA-Q190L), EGD-e C::pIMC3*kan *(InlA-T164A/K301I/G303E) or EGD-e D::pIMC3*kan *(InlA-S173I/L185F/L188I) as described in C. The invasion levels were significantly (p < 0.005) different than EGD-e InlA^m^* for all competed strains.

**Figure 8:**
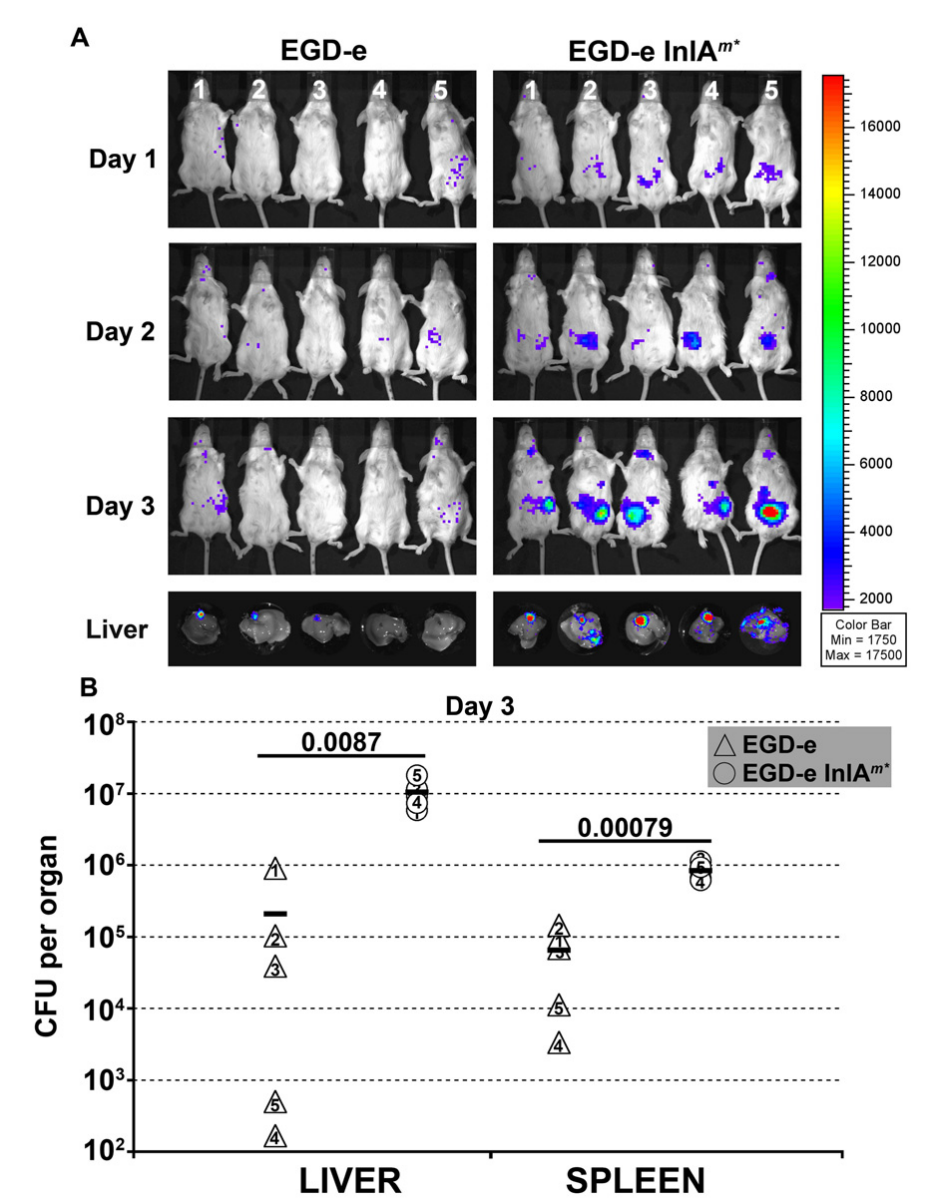
Bioluminescent imaging (BLI) of Balb/c mice orally infected with either EGD-e or EGD-e InlA^m^* (tagged with pIMK2*lux*). **A**. Balb/c mice (five per group) were gavaged with a total of 5 × 10^9 ^cfu and the progression of infection in each mouse (labelled 1 thru 5) followed on day one, two and three by BLI. Pseudocolor overlay represents the light emission profile from the infected mice with the scale bar on the right hand side. On day three mice were euthanized and livers examined *ex vivo *by BLI. **B**. Total bacterial loads from livers and spleens were numbered. The cross line denotes the mean organ cfu recovery for the five mice. Statistical analysis was conducted using a student t test with the p-value shown on the graph.

Four *inlA *sequences conferring enhanced invasion into CT-26 cells were selected to be re-created in the chromosome of *L. monocytogenes *EGD-e. The mutations constituted two single aa changes for EGD-e A (Asn259Tyr) and EGD-e B (Gln190Leu). While three aa changes were introduced into EGD-e C (T164A, K301I, Q303E) and EGD-e D (S173I, L185F, L188I). These mutations were chosen based on the frequency of isolation in *L. lactis *(EGD-e B and C), the ability to attribute the phenotype to an aa change (EGD-e A) and the isolation of mutations all confined within one LRR (EGD-e D). A fifth strain was also created based on the Lmo-InlA^m ^mutation [18], except with *Listeria *optimized codons for 192Asn and 369Ser, and was used as a positive control (EGD-e InlA^m^*). Sequencing confirmed the integrity of the newly introduced mutations, with equivalent levels of InlA expressed on the surface of the strains as compared to EGD-e (assessed by western blot - data not shown). InlA^m ^strain (termed EGD-e InlA^m^*) was compared to the parental EGD-e strain for invasion into Caco-2 and CT-26 monolayers. No differences in invasion (Figure 7a) or adherence (data not shown) were observed to Caco-2 cells, while the invasion of EGD-e InlA^m^* was significantly higher than EGD-e into CT-26 cells. We then compared the virulence of EGD-e and EGD-e InlA^m^* by competitive index (CI) assays via the intravenous (i.v.) (Figure 7b) or intragastric (i.g.) (Figure 7c) route in Balb/c mice. For i.v. inoculated mice, no differences in the kinetics of infection were observed for either strain (Figure 7b). This confirms that the two amino acid changes in InlA^m ^do not impact on the virulence of EGD-e InlA^m^* once the gastrointestinal tract is bypassed. However, EGD-e InlA^m^* was significantly more virulent when infected by the i.g. route, with higher counts obtained from livers and spleens and a significantly higher CI value (p < 0.001) for both day two (Liver 28.9, Spleen 10.6) and day three (Liver 24.9, Spleen 7.7 - Figure 7c). Neither strain was recovered form the liver nor spleen at day one post infection. Subsequent competitive index experiments were conducted by the i.g. route comparing EGD-e InlA^m^* against the strains expressing the InlA mutations identified by the CT-26 cell screen (Figure 7d). Of the four recreated strains, only EGD-e A (N259Y) gave a higher CI than EGD-e in the liver (0.19 vs 0.05) whereas identical values (0.12) were obtained for the spleens. Further experimentation will be required to access the contribution of the N259Y mutation, and it would be intriguing to see if the recombination of this mutation into EGD-e InlA^m^* would further enhance murine pathogenicity. It is interesting to note that the strain in which InlA^m ^(with *Listeria *optimized codons for 192Asn and 369Ser) was recreated (EGD-e InlA^m^* ) did not exhibit enhanced invasion or adhesion to Caco-2 cells, which is a marker for human virulence, in contrast to the previously published results [17]. To further explore the progression of i.g. infection, we repeated the Balb/c inoculations with either EGD-e or EGD-e InlA^m^* tagged with a constitutive bioluminescent *lux *marker and mice were imaged for bioluminescence on each subsequent day [18]. The EGD-e InlA^m^* strain exhibited uniform clinical signs of *L. monocytogenes *infection by day 2 [28], while these characteristics were absent from the EGD-e group even prior to sacrifice at day 3. Consistent with the clinical scores very little light was observed from the EGD-e group, while increasing light levels were obtained from the EGD-e InlA^m^* group on days 1 and 2, with a distinct foci evident in the abdomen in all 5 mice by day 3 (Figure 8a). Upon *ex vivo *imaging of the livers, a low signal was present in the gall bladder in 3 of the 5 EGD-e infected mice, whereas a much stronger signal was found from the gall bladders of all EGD-e InlA^m^* (5 out of 5) infected mice, with infection across the liver also observed (Figure 8a). The EGD-e InlA^m^* infected gall bladders were also found to be to twice the size of the EGD-e group. Further work is necessary to determine the exact extent of gall bladder colonization in these animals relative to hepatocyte infection. Enumeration of the livers and spleens confirmed that the EGD-e InlA^m^* strain produced highly reproducible i.g. infections, with the levels recovered comparable to day three i.v. infections in the liver (Figure 8b). A much larger degree of variation was observed in the EGD-e group, with statistically significant differences in bacterial counts observed between the two strains (Figure 8b). The mechanism of gall bladder colonization is currently unknown [29,30] and warrants further investigation. The EGD-e InlA^m^* strain is capable of establishing highly reproducible colonization of the gall bladder upon i.g. inoculation. This strain will be extremely useful in examining factors required for gastrointestinal transit and gall bladder colonization.

## Discussion

It is now well established that the murine model of listeriosis is limited by a poor interaction between the bacterial invasion protein InlA and its host ligand mCDH1. This is in direct contrast, to the efficient interaction between InlA and hCDH1. The discrepancy is due to a glutamate at residue 16 in mouse (and rat) E-cadherin rendering these host species relatively resistant to infection by the oral route and limiting their use as laboratory models for certain *L. monocytogenes-*mediated disease processes [11]. Recent studies have developed an engineered mouse strain expressing 'humanized' E-cadherin for studies of oral and fetoplacental listeriosis [14]. An alternative approach has utilized structure-based engineering to 'murinize' the bacterial InlA protein in order to increase affinity for murine E-cadherin [17]. This approach has provided key insights into the interaction between InlA and CDH1. While murinization was highly successful, we reasoned that additional points of contact may also improve the interaction with mCDH1. We therefore developed a system to select random mutations in InlA that enhance invasion of murine cells in order to identify novel amino acid interactions and to determine if 'murinization' of the strain can be improved.

*L. lactis *was used as a surrogate host for this process in order to prevent generation of *Listeria *mutants with increased affinity for human cells. Previous research had shown that heterologous expression of InlA from the native P*inlA *promoter in *L. lactis *could stimulate invasion into cultured human colonic enterocytes and guinea pig enterocytes in an oral infection model [27]. Additional properties of *L. lactis *such as high transformation efficiency (4 × 10^4 ^cfu for ligations) allowed us to generate multiple random libraries of substantial size and enabled the direct transformation of SDM constructs. Also the nisin inducible system enabled a high level of InlA expression on the surface of *L. lactis *in a background with relatively few sortase A anchored proteins.

The ability of *L. lactis *InlA^m^* to facilitate uptake into murine cells encouraged us to use multiple rounds of *en masse *enrichment of InlA mutant libraries through CT-26 cells. The cumulative results from each passage showed a continued improvement in the invasion efficiency, suggestive of an enrichment of positive clones. A surprising level of diversity in InlA clones was apparent (across the 4 banks) with 25 of the 32 clones analyzed exhibiting unique sequences. Only bank iii with the lowest frequency of mutations exhibited a degree of clonality (4/8 were Q190L). This suggests that we have not yet uncovered the full complement of mutations within the banks which confer enhanced invasion capabilities.

Directed evolution of the *inlA *gene has the potential to uncover mutations not predicted by a structure-based approach (Table 2). With respect to the Q190L mutation the glutamine at residue 190 found on LRR 6 within the hydrophobic pocket, and forms a hydrogen bond to proline 16 in hCDH1. The change to leucine may affect the pocket and improve access of glutamic acid 16 in mCDH1. Of all the single amino acid changes, the N259Y mutation exhibited the single greatest invasion increase into CT-26 cells. Combining this mutation with either T399I or L149 M was shown to reduce or enhance invasion, respectively, with the negative effect of the T399I confirmed by the reduction in invasion efficiency observed when combined with additional positive mutations (bank IV, clone 8 *versus *bank IV, clone 1-Table 2). Further biochemical studies will be required to identify the role these mutations play to enhance the interaction with mCDH1. The previously identified single aa changes at residues 192 and 369 [17] each increased invasion ~20 fold, whereas the combined 192 + 369 mutations increased invasion ~30 fold. The identical aa change at residue 369 was also isolated from our error prone PCR bank. However, this clone contained additional mutations that resulted in a reduced level of invasion compared to the 369 single mutant.

The CDH1 interacting amino acids appear to be highly conserved and recalcitrant to change [31]. From a collection of 101 *inlA *gene sequences mapped onto the InlA crystal structure [32], three naturally occurring InlA variants were identified which could potentially mediate an interaction with hCDH1, with one (Lys301Glu) also identified through the random mutagenesis approach in our study. However, while all mutants containing this residue had a positive effect on invasion into CT-26 cells, the exact contribution of this residue could not be assessed as additional mutations were present in all clones. Further analysis of individual clones from each bank or the application of additional selection is required due to the diversity uncovered (25 of the 32 clones analyzed were different). This diversity and the enhanced invasion of all the clones examined confirms that amino acids additional to the ones previously examined [17] can modulate the affinity for CDH1.

Despite the analysis of 32 clones from our enriched bank of InlA variants, we failed to detect mutations that yielded invasion rates comparable to the murinized InlA described by Wollert and coworkers [17]. In terms of developing usable models of murine listeriosis the approach of 'murinizing' the bacterial strain arguably has a number of benefits over the development of humanized mouse lines. Development of the modified bacterium will permit utilization of this strain in existing mouse lines (including existing knock-out murine models) and distribution of the murinized strain is relatively straightforward, as is the creation of new mutations in the EGD-e InlA^m^* background. However, the 2-fold enhanced adherence and invasion to human (Caco-2) cells of the *L. monocytogenes *Lmo-InlA^m ^[17] could be a potential cause for concern as it is suggestive of a slight enhancement of virulence towards humans. The procedure used to create that strain required multiple prolonged incubations at 42°C [17,33]. It has been recently shown that high temperature growth of *L. monocytogenes *can induce spontaneous mutation, suggesting that high temperature growth should be minimized to avoid the acquisition of secondary mutations [34]. We re-created the InlA mutations described by Wollert *et al*., [17] to create EGD-e InlA^m^* using only two temperature shifts to 37°C and six passages under non-selective conditions [20]. Another difference between the Lmo-InlA^m ^and EGD-e InlA^m^* strain were the nucleotide changes made to create the mutated amino acids. In the EGD-e InlA^m^* strain the two codons were chosen based on the codon usage from genome analysis, with the most commonly used triplets applied. In each case usage was 50% higher than the one used in Lmo-InlA^m^. For the asparagine 192, AAT compared to the AAC codon was chosen (31.8 vs 14.4 per 1000 codons). While for serine 369 TCT compared to TCG codon was chosen (12.8 vs 6.2 per 1000 codons). The invasion data for Lmo-InlA^m ^agreed with the biophysical characterization which showed an enhanced interaction for InlA with CDH1 [35] however as recently shown, synonymous mutations leading to mRNA sequence changes can also affect substrate specificity or protein activity [36]. To access the role of codon usage or strain background, competitive index experiments will need to be conducted to directly compare Lmo-InlA^m ^with EGD-e InlA^m^*.

## Conclusions

The research presented here generated random InlA variants with enhanced invasion into the CT-26 cell line most likely through an increased affinity for mCDH1. Novel mutations in InlA were readily identified from the random mutagenesis approach and a number (including the N259Y mutation) are worthy of further study. The approach used here indicates that other random or targeted mutagenesis strategies may uncover mutations that further enhance protein-ligand binding. In particular we suggest that screening approaches such as biopanning [37] using the first extra cellular domain of mCDH1 as bait or a site-saturation mutagenesis approach (the analysis of all amino acid combinations at a single residue) [38] may uncover further potential interactions. We have demonstrated that the newly created strain, EGD-e InlA^m^* does not have an enhanced affinity for human cells (unlike the predecessor EGD-InlA^m^) while displaying highly reproducible oral infections in the mouse model. The use of this murinized *L. monocytogenes *strain will prove a useful tool in analysing the gastrointestinal phase of listeriosis. The additional residues identified here as playing a role in InlA::CDH1 interactions will inform our ongoing efforts to create safer 'murinised' versions of *L. monocytogenes *which will help us to combat this often fatal pathogen.

## Authors' contributions

All authors read and approved the final manuscript. IRM devised the study, carried out the experimental work and wrote the manuscript; PGC carried out murine infection work; CH and CGMG devised and guided the study and helped to draft the manuscript.
